# Perioperative Management and Outcomes of Open Aortic Aneurysm Repair in Ethiopia

**DOI:** 10.7759/cureus.93560

**Published:** 2025-09-30

**Authors:** Ananya Abate Shiferaw, Tensaye Woldemichael

**Affiliations:** 1 Anesthesia and Critical Care, University of Botswana Faculty of Medicine, Gaborone, BWA; 2 Anesthesiology, Addis Ababa University College of Health Sciences, Addis Ababa, ETH

**Keywords:** anesthesia, aortic aneurysm repair, cardiothoracic, low- and middle-income countries, open aortic aneurysm repair, resource-limited setting, surgery, tikur anbessa specialized hospital (tash), vascular

## Abstract

Background

Aortic aneurysms (AAs) are life-threatening conditions requiring timely surgical intervention. While endovascular repair is most common in high-income countries, open surgical repair (OSR) remains the only option in low-resource settings like Ethiopia. This study aimed to examine patient characteristics, perioperative management, and outcomes of OSR at Tikur Anbessa Specialized Hospital (TASH) in Addis Ababa, Ethiopia.

Methods

We conducted a retrospective observational study of adult patients who underwent OSR between January 2017 and May 2024. Data were extracted from the operating room (OR) logbook and surgical intensive care unit (SICU) registry using a structured tool. Descriptive statistics summarized patient characteristics and perioperative care, while logistic regression analyses identified predictors of in-hospital mortality and major complications. Statistical significance was set at p < 0.05.

Results

A total of 45 patients (mean age 54.4 ± 16.3 years; 57.8% female) were included in the study. Most had abdominal aneurysms (88.9%) and underwent elective surgery. In-hospital mortality was 13.3%, and 37.8% experienced major complications, excluding deaths. Acute kidney injury (AKI, 22.2%) and hospital-acquired pneumonia (15.8%) were the most common. Ischemic heart disease (IHD) was notably associated with increased risk of mortality (crude odds ratio (COR) = 9.25; p = 0.080), AKI (COR = 14.57; p = 0.030), and respiratory complications (COR = 27.75; p = 0.009). A cross-clamp time exceeding 90 minutes was significantly associated with increased mortality (COR = 7.75; p = 0.035) and AKI (COR = 7.25; p = 0.013). Elevated baseline serum creatinine (≥1 mg/dL) was also significantly associated with the development of AKI (COR = 22.67; p = 0.006).

Conclusion

This study highlights the feasibility and challenges of open AA repair in resource-limited Ethiopia. Despite a few cases, younger, low-comorbidity patients reflect selective surgery due to constraints. Shortages in equipment, medications, and ICU beds significantly hindered perioperative monitoring, anesthesia care, and pain management, impacting overall surgical outcomes.

## Introduction

An aortic aneurysm (AA) is an abnormal dilation of a segment of the aorta, defined as an increase in diameter of at least 50% compared to the normal expected size [1]. AAs are classified based on size, location, shape, and underlying cause, with abdominal AAs (AAAs) being the most common type, accounting for the majority of cases [2]. The incidence of AAs is rising globally, driven by aging populations and risk factors such as smoking [3, 4]. Despite advancements in diagnostic techniques and treatment modalities, AAs remain a significant health threat due to their high mortality rates. Ruptured AAAs have an alarming mortality rate of 40% to 80%, while data from high-income countries show that the mortality rate for elective AAA repairs is around 3% to 8%, underscoring the critical need for effective prevention and management strategies [5-9].

While endovascular aneurysm repair (EVAR) has become the most common treatment option for aortic aneurysms in many developed countries, open surgical repair (OSR) is increasingly reserved for complex cases [10, 11]. However, in developing countries like Ethiopia, where EVAR facilities are not yet available, open surgery remains the primary treatment option for all AAs [12]. Data regarding perioperative care and outcomes of open AA repair are lacking in Ethiopia. This study aimed primarily to describe the patient characteristics, perioperative management, and in-hospital outcomes of open AA repair at Tikur Anbessa Specialized Hospital (TASH), Addis Ababa, Ethiopia, between 2017 and 2024, and secondarily to explore associations between selected clinical and intraoperative factors and key outcomes. An enhanced understanding of the complex procedure in a low-income setting may improve the care of patients undergoing open AA repair in Ethiopia. 

## Materials and methods

Study design, area, and period

The study was a single-center retrospective observational study. It was conducted at TASH, the largest referral hospital in Addis Ababa, Ethiopia, with 800 beds. TASH serves as the primary teaching facility for clinical and preclinical training in most disciplines and provides specialized clinical services not available in other public or private institutions in the country. The hospital has a mixed adult ICU where six beds are reserved for surgical and trauma patients and six beds for medical patients. The human resource profile included three vascular surgeons, three fellows, two cardiac anesthesiologists, one cardiac anesthesia fellow, and 14 general anesthesiologists. The study period covered cases from January 2017 to May 2024.

Inclusion and exclusion criteria

The study population included all patients aged ≥18 years who underwent open AA repair during the study period. Patients with incomplete medical records were excluded.

Sampling procedure

Given the rarity of open AA repair in this setting, the study employed consecutive sampling of all eligible cases performed during the study period.

Study variables

Dependent variables were in-hospital mortality and major complications (morbidity), including acute kidney injury (AKI), myocardial infarction (MI), bowel ischemia, arrhythmia, paraplegia, respiratory complications, and thromboembolism.

Independent variables were demographics (age and sex), clinical characteristics (comorbidities and preoperative medications), aneurysm characteristics (type, site, and size of the aneurysm), and intraoperative factors (blood loss, cross-clamp time, duration of surgery, intraoperative fluids and blood products, intraoperative hemodynamics, and intraoperative drugs).

Operational definitions

In-Hospital Mortality

This included* *any death occurring postoperatively before discharge from the hospital.

Major Complication (Morbidity)

The occurrence of one or more postoperative complications, including AKI, MI, arrhythmia, paraplegia, respiratory complications (defined as postoperative respiratory events, including hospital-acquired pneumonia, respiratory failure, pulmonary edema, atelectasis, acute respiratory distress syndrome (ARDS), etc.), mesenteric ischemia, or thromboembolism (including deep venous thrombosis (DVT) and pulmonary embolism (PE)), as documented in the patient’s progress notes by the treating physician.

Data collection procedure

Patient medical records were identified from the operating room (OR) logbook and the surgical intensive care unit (SICU) register. Data were collected using a structured data extraction tool. Anonymity maintained.

Data analysis procedure

The collected data were checked for completeness and entered in IBM SPSS Statistics for Windows, version 27.0 (IBM Corp., Armonk, NY). Descriptive statistics were performed to summarize the socio-demographic and clinical characteristics of the participants and perioperative care. Binary logistic regressions were conducted for each predictor variable and categorical outcome variable. A p-value of less than 0.05 was considered statistically significant.

Ethical clearance

Ethical clearance was obtained from the Research and Ethics Committee of the Department of Anesthesiology, Addis Ababa University, on June 25, 2024 (approval no. ACCPM01/2024)

## Results

A total of 50 patients were identified from the OR logbook and SICU register during the study period. Of these, five patients were excluded due to incomplete data, leaving a final sample size of 45 patients included in the analysis.

Demographics and preoperative characteristics

The study population was predominantly female and relatively young, with a median age of 58 years (range: 17-76 years). Most patients were generally healthy, with few identified as smokers. Chronic hypertension was the most common comorbidity. Among preoperative medications, beta-blockers were most frequently used, followed by calcium channel blockers, aspirin, and statins (Table 1).

**Table 1:** Demographics and Preoperative Characteristics of Study Participants (N=45) Data are presented as n (%a) or mean ± SD. SD: standard deviation; n: number of participants Unit: mg/dL = milligrams per deciliter

Variable		Frequency n (%)
Gender	Male	19 (42.2%)
	Female	26 (57.8%)
Age	>65 years	15 (33.3%)
	Mean age (in years)	54.44 ± 16.34 years
Total number of patients with one or more comorbidities		29 (64.4%)
Hypertension, n (%)		22 (48.9%)
Valvular heart disease, n (%)		6 (13.3%)
Ischemic heart disease (IHD), n (%)		4 (8.9%)
Diabetes mellitus, n (%)		2 (4.4%)
Heart failure, n (%)		2 (4.4%)
Baseline creatinine > 1mg/dl		2 (4.4%)
Smokers, n (%)		3 (6.7%)
Preoperative medications	Beta-blockers	36 (80%)
	Calcium channel blockers	15 (33.3%)
	Aspirin and statins	11 (24.4%)

Characteristics of the aneurysm

AAAs were the most common type, with infrarenal aneurysms representing the predominant subtype. Thoracic AAs (TAAs) were less frequent, comprising ascending (n=2, 4.4%) and descending (n=3, 6.7%) TAAs. The mean aneurysm diameter was 6.6 ± 2.3 cm (range: 2.7-18 cm). Most patients underwent elective repair. Ruptured AAs were more common in male patients (n=3/19, 15.7%) than in female patients (n=2/26, 7.6%) (Table 2).

**Table 2:** Aneurysm Characteristics and Type of Surgery (N=45) Data are presented as n (%); n = number of participants

Variable			Frequency n (%)
Type of aneurysm	Abdominal	Infrarenal subtype	36 (80%)
		Suprarenal subtype	4 (8.9%)
		Total	40 (88.9%)
	Thoracic		5 (11.1%)
Type of surgery	Elective		40 (88.9%)
	Emergency		5 (11.1%)

Intraoperative care

All surgeries were performed under general anesthesia using endotracheal intubation. Commonly used induction agents included propofol (n=33, 73.3%), ketamine (n=8, 17.8%), and thiopental (n=4, 8.9%). Thiopental was used when propofol was not available. The other induction agent, etomidate, was unavailable. Fentanyl (n=34, 75.6%) and morphine (n=10, 22.2%) were the primary opioids used during induction and maintenance. Few patients received intravenous paracetamol (n=2, 4.4%) and epidural bupivacaine (n=3, 6.7%) as part of multimodal analgesia. One patient received tramadol as a sole analgesic agent. Vecuronium (n=33, 76.4%), succinylcholine (n=10, 22.2%), and pancuronium (n=1, 2.2%) were the muscle relaxants used for induction. Vecuronium (n=43, 95.6%) was the most commonly used muscle relaxant for maintenance, followed by pancuronium (n=2, 4.4%). Cisatracurium, atracurium, and rocuronium were not readily available. Isoflurane (n=39, 86.7%) was the preferred inhalational agent for anesthesia maintenance as compared to the other available agent, halothane. Sevoflurane was not available. 

Intraoperative monitoring included ECG, non-invasive blood pressure (NIBP), and SpO₂. Arterial lines were used in 82.2% (n=37) of patients, while central venous catheters were placed in 17.8% (n=8). Temperature and end-tidal carbon dioxide (ETCO₂) were not monitored, as the operating room monitors lacked the necessary accessories for these parameters.

The mean duration of surgery and anesthesia was approximately four and five hours, respectively, with mean aortic cross-clamp times exceeding one hour in a quarter of patients. Blood loss was substantial, often requiring transfusion, and crystalloid use was high. Hemodynamic variability was considerable, necessitating vasopressors in the majority of patients (n=37, 82.2%) (Table 3). Noradrenaline (n=33, 73.3%) and adrenaline (n=11, 24.4%) were the vasopressors used. Seven patients (15.6%) required the use of double vasopressors. Antihypertensives used were nitroglycerine (n=14, 31.1%) and labetalol (n=6, 13.3%). Additional drugs included atropine (n=5, 11.1%) and mannitol (n=22, 48.9%). All patients received heparin intraoperatively. 

**Table 3:** Summary of Intraoperative Care of Study Participants Data are presented as mean ± SD or n (%). SD: standard deviation; n: number of participants; PRBCs: packed red blood cells; MAP: mean arterial blood pressure; bpm: beats per minute. Units: ml = milliliter; ml/kg/hr = milliliters per kilogram per hour; mmHg = millimeters of mercury

Variable		Mean ± SD or n (%)
Duration of surgery (minutes)		245 ± 74 (Range: 90-420 minutes)
Duration of anesthesia (minutes)		288 ± 80 (Range: 120-480 minutes)
Aortic cross-clamp time (minutes)		76 ± 29 (Range: 30-160 minutes)
Cross-clamp time >90 minutes, n (%)		12 (26.7%)
Estimated blood loss (ml)		1567 ± 1174 (Range: 50-5400 ml)
Blood transfusion, n (%)		39 (86.7%)
PRBC transfusion (units), mean ± SD		2.5 ± 1.7
Crystalloid infusion (ml), mean ± SD		3321 ± 1380 (Range: 500-7000 ml)
Intraoperative urine output (ml/kg/h), mean ± SD		2.7 ± 1.6 (Range: 0.4 – 5.9 ml/kg/h)
Cardiovascular parameters, mean ± SD	Highest heart rate	97 ± 15 (Range: 65-130 bpm)
	Lowest heart rate	69 ± 15 (Range: 40-98 bpm)
	Highest systolic blood pressure	142 ± 20 (Range: 100 – 200 mmHg)
	Lowest systolic blood pressure	85 ± 10 (Range: 70 – 110 mmHg)
	Highest diastolic blood pressure	82 ± 13 (Range: 60 – 120 mmHg)
	Lowest diastolic blood pressure	54 ± 7.5 (Range: 40 – 80 mmHg)
	Highest MAP	95 ± 15 (Range: 60 – 130 mmHg)
	Lowest MAP	60 ± 7 (Range: 45 – 75 mmHg)

Postoperative care

All patients were transferred to the ICU postoperatively, with 22.2% (n=10) arriving intubated. All thoracic aneurysm repairs required postoperative mechanical ventilation. The mean duration of mechanical ventilation was 0.8 ± 2.6 days (range: 0-17 days). Most patients (n=22, 55.6%) received parenteral morphine and oral paracetamol for analgesia, while 10 patients (22.2%) received fentanyl instead of morphine. Epidural analgesia with bupivacaine was used in only three patients (6.7%), mainly due to the limited availability of epidural kits at TASH. 

Outcomes

In-hospital mortality was 13.3% (n=6). Mortality was higher among male patients (n=4/19, 21.1%) compared with female patients (n=2/26, 7.6%). No difference in mortality rate is observed between the age group > 65 years (n=2/15, 13.3%) and those <65 years (n=4/30, 13.3%). Major complications occurred in 37.8% (n=17) of patients. The most common complications were AKI (n=10, 22.2%) and hospital-acquired pneumonia (n=7, 15.8%). Less common complications included MI (n=2, 4.4%), DVT (n=2, 4.4%), mesenteric ischemia (n=1, 2.2%), and pulmonary thromboembolism (n=1, 2.2%).

Factors associated with mortality and morbidity

Due to the small sample size and the limited number of in-hospital deaths (n = 6), multivariate logistic regression models produced unstable estimates and failed to converge, even when penalized methods such as Firth logistic regression were applied. Therefore, bivariate analysis was conducted using Fisher’s exact test, which is appropriate for small samples and sparse data. Crude odds ratios (CORs) with 95% confidence intervals (CI) were calculated to assess associations between selected clinical variables and in-hospital mortality, AKI, and respiratory complications. Among the variables examined, ischemic heart disease (IHD) showed a notable association with mortality (COR = 9.25; 95% CI: 1.01-84.73; p = 0.080), and prolonged cross-clamp time (>90 minutes) was significantly associated with increased mortality (COR = 7.75; 95% CI: 1, 1.20-50.13; p = 0.035). Other variables such as age, sex, hypertension, baseline creatinine, and type of surgery did not show statistically significant associations (Table 4).

**Table 4:** Bivariate Logistic Regression Analysis of Association between Clinical Variables and In-Hospital Death (N = 45) Data are presented as n (%), where n = number of participants and percentages are row percentages. Odds ratios (ORs) are reported as crude OR (COR) with 95% confidence interval (CI). Statistical significance was assessed using Fisher’s exact test. *A p-value < 0.05 was considered statistically significant. Variables with very few cases were collapsed where appropriate to enhance interpretability. Units: mg/dL = milligrams per deciliter; ml = milliliters

Variable		In-Hospital Death n (%)		COR (95% CI)	Fisher’s exact p-value
		No	Yes		
Age	18 – 65 years	26 (86.7%)	4 (13.5%)	1	1
	>65 years	13 (86.7%)	2 (13.5%)	1.00 (0.16, 6.19)	1.000
Sex	Male	15 (78.9%)	4 (21.1%)	1	1
	Female	24 (92.3%)	2 (7.7%)	0.31 (0.05, 1.92)	0.377
Hypertension	No	22 (95.7%)	1 (4.3%)	1	1
	Yes	17 (77.3%)	5 (22.7%)	6.47 (0.69, 60.68)	0.096
Ischemic heart disease	No	37 (90.2%)	4 (9.8%)	1	1
	Yes	2 (50.0%)	2 (50.0%)	9.25 (1.01, 84.73)	0.080
Smoking	No	37 (88.1%)	5 (11.9%)	1	1
	Yes	2 (66.7%)	1 (33.3%)	3.70 (0.28, 48.62)	0.356
Baseline creatinine	< 1 mg/dl	36 (90.0%)	4 (10.0%)	1	1
	>= 1 mg/dl	3 (60.0%)	2 (40.0%)	6.00 (0.76, 47.36)	0.125
Blood loss	<=1000 ml	18 (90.0%)	2 (10.0%)	1	1
	>1000 ml	21 (84.0%)	4 (16.0%)	1.71 (0.28, 10.48)	0.678
Blood transfusion	No	5 (83.3%)	1 (16.7%)	1	1
	Yes	34 (87.2%)	5 (12.8%)	0.74 (0.07, 7.66)	1.000
Component transfusion	No	29 (90.6%)	3 (9.40%)	1	1
	Yes	10 (76.9%)	3 (23.1%)	2.90 (0.50, 16.76)	0.334
Site of the aortic aneurysm	Thoracic	3 (60.0%)	2 (40.0%)	1	1
	Abdominal	36 (90.0%)	4 (10.0%)	0.17 (0.02, 1.31)	0.125
Cross-clamp time	<=90 minutes	31 (93.9%)	2 (6.10%)	1	1
	>90 minutes	8 (66.7%)	4 (33.3%)	7.75 (1.20, 50.13)	0.035*
Type of surgery	Elective	36 (90.0%)	4 (10.0%)	1	1
	Emergency	3 (60.0%)	2 (40.0%)	6.00 (0.76, 47.36)	0.125
Surgical duration	<=240 minutes	22 (91.7%)	2 (8.30%)	1	1
	>240 minutes	17 (81.0%)	4 (19.0%)	2.59 (0.42, 15.84)	0.396

For AKI, significant associations were observed with IHD (COR = 14.57; 95% CI: 1.32-161.42; p = 0.030), elevated baseline creatinine (≥1 mg/dL; COR = 22.67; 95% CI: 2.15-239.32; p = 0.006), and cross-clamp time >90 minutes (COR = 7.25; 95% CI: 1.55-33.84; p = 0.013) (Table 5). Similarly, respiratory complications were significantly associated with IHD (COR = 27.75; 95% CI: 2.31-333.76; p = 0.009) (Table 6). No other variables demonstrated statistically significant associations with AKI or respiratory complications.

**Table 5:** Bivariate Logistic Regression Analysis of Association Between Clinical Variables and Acute Kidney Injury (N = 45) Data are presented as n (%), where n = number of participants and percentages are row percentages. ORs are reported as crude OR (COR) with 95% confidence interval (CI). Statistical significance was assessed using Fisher’s exact test. *A p-value < 0.05 was considered statistically significant. Variables with very few cases were collapsed where appropriate to enhance interpretability. Units: mg/dL = milligrams per deciliter; ml = milliliters

Variable		Acute kidney injury n (%)		COR (95% CI)	Fisher’s exact p-value
		No	Yes		
Age	18 – 65 years	25 (83.3%)	5 (16.7%)	1	1
	>65 years	10 (66.7%)	5 (33.3%)	0.26 (0.59, 10.56)	0.253
Sex	Male	13 (68.4%)	6 (31.6%)	1	1
	Female	22 (84.6%)	4 (15.4%)	0.39 (0.09, 1.66)	0.281
Hypertension	No	19 (82.6%)	4 (17.4%)	1	1
	Yes	16 (72.7%)	6 (27.3%)	1.78 (0.43, 7.44)	0.491
Ischemic heart disease	No	34 (82.9%)	7 (17.1%)	1	1
	Yes	1 (25.0%)	3 (75.0%)	14.57 (1.32, 161.42)	0.030*
Smoking	No	34 (81.0%)	8 (19.0%)	1	1
	Yes	1 (33.3%)	2 (66.7%)	8.50 (0.68, 105.75)	0.119
Baseline creatinine	< 1 mg/dl	34 (85.0%)	6 (15.0%)	1	1
	>= 1 mg/dl	1 (20.0%)	4 (80.0%)	22.67 (2.15, 239.32)	0.006*
Blood loss	<=1000 ml	18 (90.0%)	2 (10.0%)	1	1
	>1000 ml	17 (68.0%)	8 (32.0%)	4.24 (0.79, 22.85)	0.147
Component transfusion	No	27 (84.4%)	5 (15.6%)	1	1
	Yes	8 (61.5%)	5 (38.5%)	3.38 (0.78, 14.67)	0.124
Site of the aortic aneurysm	Thoracic	4 (80.0%)	1 (20.0%)	1	1
	Abdominal	31 (77.5%)	9 (22.5%)	1.15 (0.12, 11.74)	1.000
Cross-clamp time	<=90 minutes	29 (87.9%)	4 (12.1%)	1	1
	>90 minutes	6 (50.0%)	6 (50.0%)	7.25 (1.55, 33.84)	0.013*
Type of surgery	Elective	31 (77.5%)	9 (22.5%)	1	1
	Emergency	4 (80.0%)	1 (20.0%)	0.86 (0.09, 8.71)	1.000
Surgical duration	<=240 minutes	21 (87.5%)	3 (12.5%)	1	1
	>240 minutes	14 (66.7%)	7 (33.3%)	3.50 (0.77, 15.88)	0.151

**Table 6:** Bivariate Logistic Regression Analysis of Association between Clinical Variables and Respiratory Complications (N = 45) Data are presented as n (%), where n = number of participants and percentages are row percentages. Odds ratios (ORs) are reported as crude OR (COR) with 95% confidence interval (CI). Statistical significance was assessed using Fisher’s exact test. *A p-value < 0.05 was considered statistically significant. Variables with very few cases were collapsed where appropriate to enhance interpretability. Units: mg/dL = milligrams per deciliter; ml = milliliters

Variable		Respiratory complication n (%)		COR (95% CI)	Fisher’s exact p-value
		No	Yes		
Age	18 – 65 years	26 (86.7%)	4 (13.3%)	1	1
	>65 years	12 (80.0%)	3 (20.0%)	1.63 (0.31, 8.43)	0.670
Sex	Male	15 (78.9%)	4 (21.1%)	1	1
	Female	23 (88.5%)	3 (11.5%)	0.49 (0.09, 2.50)	0.433
Hypertension	No	20 (87.0%)	3 (13.0%)	1	1
	Yes	18 (81.8%)	4 (18.2%)	1.48 (0.29, 7.54)	0.699
Ischemic heart disease	No	37 (90.2%)	4 (9.8%)	1	1
	Yes	1 (25.0%)	3 (75.0%)	27.75 (2.31, 333.76)	0.009*
Smoking	No	36 (85.7%)	6 (14.3%)	1	1
	Yes	2 (66.7%)	1 (33.3%)	3.00 (0.23, 38.47)	0.405
Asthma	No	35 (85.4%)	6 (14.6%)	1	1
	Yes	3 (75.0%)	1 (25.0%)	1.94 (0.17, 21.94)	0.505
Baseline creatinine	< 1 mg/dl	35 (87.5%)	5 (12.5%)	1	1
	>= 1 mg/dl	3 (60.0%)	2 (40.0%)	4.67 (0.62, 35.17)	0.166
Blood loss	<=1000 ml	17 (85.0%)	3 (15.0%)	1	1
	>1000 ml	21 (84.0%)	4 (16.0%)	1.08 (0.21, 5.50)	1.000
Site of aortic aneurysm	Thoracic	4 (80.0%)	1 (20.0%)	1	1
	Abdominal	34 (85.0%)	6 (15.0%)	0.71 (0.07, 7.45)	1.000
Cross-clamp time	<=90 minutes	29 (87.9%)	4 (12.1%)	1	1
	>90 minutes	9 (75.0%)	3 (25.0%)	2.42 (0.45, 12.88)	0.362
Type of surgery	Elective	34 (85.0%)	6 (15.0%)	1	1
	Emergency	4 (80.0%)	1 (20.0%)	1.41 (0.13, 14.96)	1.000
Surgical duration	<=240 minutes	22 (91.7%)	2 (8.3%)	1	1
	>240 minutes	16 (76.2%)	5 (23.8%)	3.44 (0.59, 20.02)	0.225

## Discussion

This study provided valuable insights into the demographic, perioperative care, and outcome of patients undergoing AA repair in a resource-limited setting in Ethiopia. AA repair surgery was performed infrequently in our setup, with a relatively younger patient population and a low prevalence of comorbidities, including smoking, compared to high-income countries. The average aneurysm size was larger than that reported in other studies. Perioperative care was constrained by limited resources, including manpower, monitoring equipment, anesthesia, and surgical supplies, as well as limited ICU beds. Additionally, optimal pain management was not fully implemented, with very few patients receiving epidural analgesia. There was also less use of preoperative medications, such as aspirin and statins, compared to other settings. As a result, mortality rates in our cohort were higher than those reported in studies of AAA repair in high-income countries [13-17]. IHD and prolonged aortic cross-clamp time were associated with increased mortality and morbidity. The most common complications observed were AKI and respiratory issues.

This study showed that the procedure is infrequently performed, with only 50 cases over a seven-year period. This limited number of cases can likely be attributed to resource constraints, including a small healthcare workforce and the unavailability of essential supplies. For instance, arterial lines were used in 82.2% (n=37) of patients, while central venous catheters were placed in only 17.8% (n=8) of cases. Additionally, none of the patients had temperature and EtCO₂ monitoring during surgery. Medications like etomidate, sevoflurane, and crucial muscle relaxants such as rocuronium, atracurium, and cisatracurium were unavailable. These factors suggest that many patients might have been deferred from surgery due to the lack of necessary resources, contributing to the low procedural volume in our setting. Lost medical records may have also contributed to this. As far as we are aware, during the study period, TASH was the only center in Ethiopia providing open AA repair. Given this, the surgical volume in our cohort likely reflects national capacity rather than merely institutional practice.

Our cohort was notably younger, with a mean age of 54.7 ± 16 years, compared to the 72-77 years typically seen in high-income countries [5, 6]. The age distribution in our study aligns more closely with data from a systematic review of AA patterns in Africa, where the mean age was 56 years [18]. This younger demographic, alongside the higher prevalence of female patients (57.8%), may be reflective of a more selective surgical approach in our setting. Given the limitations in healthcare resources, it is likely that only patients with a high likelihood of survival were chosen for surgery, potentially leading to the exclusion of older individuals and those with significant comorbidities. This selective approach could explain the younger age and differing comorbidity profiles observed in our cohort.

Regarding preoperative comorbidities, chronic hypertension was the most common (n=22, 48.9%), although this was lower than the 82% reported in high-income countries [5, 6]. This lower incidence of hypertension may also be a reflection of the selective nature of surgical patient inclusion, with only the less risky patients being selected for surgery. Patients with multiple comorbidities or advanced age, who may have been deemed at higher risk, might have been deferred for surgery due to limited resources such as ICU beds and postoperative care facilities.

In terms of preoperative medication, beta-blockers were the most frequently used drug (n=36, 80%), followed by aspirin (n=11, 24.4%) and statins (n=11, 24.4%). This pattern was similar to other studies, although a higher proportion of patients in high-income countries were on aspirin (71%) and statins (62%) [7], likely due to a higher prevalence of cardiac comorbidities in those populations.

Intraoperatively, most surgeries were performed under general anesthesia, with isoflurane as the most commonly used inhalational agent and vecuronium as the primary muscle relaxant. The average durations of surgery (243 ± 74 minutes) and anesthesia (288 ± 80 minutes) were consistent with expectations for aortic aneurysm repair. However, the average cross-clamp time of 75.5 minutes was significantly higher than the 30-45 minutes reported in other studies [16, 19], which could contribute to the increased morbidity and mortality observed in our cohort. This extended cross-clamp time may also be related to the more limited technical support and equipment available during surgeries, which could complicate the procedure.

Postoperatively, all patients were admitted to the ICU, with mechanical ventilation required for an average of 0.7 days. The mean ICU stay of 4.07 days was comparable to that found in other studies [8]. However, resource constraints may have limited the ability to provide optimal care in the ICU, as seen in the relatively small number of patients who received more advanced pain management interventions. Only a few patients (n=3, 6.7%) received epidural analgesia with bupivacaine for pain control, a deficiency that may have contributed to the higher incidence of respiratory complications (n=7, 15.6%) observed in our cohort as compared to reports in other studies [15].

The in-hospital mortality rate of 13.3% (n=6) observed in this study was higher than rates reported in high-income countries (2.9%-7%) [13-17], but lower than that reported in a South African study (16.6%) [8]. The incidence of AKI was 22.2% (n=10), which aligns with rates seen in elective AAA surgeries in other settings (approximately 18.9%) [15, 19, 20]. A significant association was observed between prolonged aortic cross-clamp time (>90 minutes), elevated baseline creatinine levels (≥1 mg/dL), and IHD with the development of AKI, aligning with findings from previous studies [14, 15, 19]. However, the anatomical site of the aneurysm was not associated with AKI in our cohort, which contrasts with other reports indicating higher AKI rates in patients with suprarenal clamping [14]. This discrepancy may be due to the small sample size in our study. IHD was associated with in-hospital mortality, AKI, and respiratory complications, highlighting the importance of vigilant perioperative management in this high-risk population. 

This study underscores the complexity of aortic aneurysm repair in a low-income setting, where resource limitations and a selective surgical approach contribute to variations in patient demographics and outcomes. Several limitations should be considered when interpreting these findings. First, the retrospective nature of the study introduces potential biases related to data completeness and accuracy. Second, the small sample size limited statistical power and precluded multivariate analysis, restricting the ability to adjust for potential confounders. As a result, only CORs could be reported, which may not fully reflect the independent effect of each variable. Third, the study was conducted at a single center, which may affect the generalizability of the findings to other settings with different patient populations or healthcare infrastructures. Additionally, data quality was compromised by inconsistent documentation and limited availability of post-discharge information, making it difficult to assess long-term outcomes. Detailed procedural data were not included, as most cases involved similar open techniques performed by a small group of surgeons. These limitations highlight the need for prospective, multi-center studies with robust data collection systems to better understand and improve outcomes in this high-risk population.

## Conclusions

This study highlights the unique challenges and outcomes associated with OSR of AAs in a resource-limited setting in Ethiopia. The procedure was performed infrequently, with a younger and predominantly female patient population, reflecting a selective surgical approach driven by constrained healthcare resources. Despite the lower prevalence of comorbidities, the average aneurysm size was larger, and perioperative care was significantly limited by shortages in monitoring equipment, medications, and ICU capacity. These limitations contributed to higher morbidity and mortality rates compared to high-income countries, with IHD and prolonged aortic cross-clamp time emerging as key predictors of poor outcomes. The findings underscore the urgent need for investment in perioperative infrastructure, enhanced pain management strategies, and improved access to essential surgical and anesthetic supplies. Addressing these gaps could improve patient outcomes and expand the feasibility of AA repair in similar low-resource settings.
